# Surface reconstructions and related local properties of a BiFeO_3_ thin film

**DOI:** 10.1038/srep39698

**Published:** 2017-01-19

**Authors:** L. Jin, P. X. Xu, Y. Zeng, L. Lu, J. Barthel, T. Schulthess, R. E. Dunin-Borkowski, H. Wang, C. L. Jia

**Affiliations:** 1 Peter Grünberg Institute (PGI-5), Research Centre Jülich, 52425 Jülich, Germany; 2 Ernst Ruska-Centre for Microscopy and Spectroscopy with Electrons (ER-C), Research Centre Jülich, 52425 Jülich, Germany; 3 Institute for Theoretical Physics, ETH Zurich, 8093 Zurich, Switzerland; 4 The School of Electronic and Information Engineering, Xi’an Jiaotong University (XJTU), Xi’an 710049, China; 5 State Key Lab of New Ceramics and Fine Processing and School of Materials Science and Engineering, Tsinghua University, Beijing 100084, China; 6 Central Facility for Electron Microscopy, RWTH Aachen University, 52074 Aachen, Germany; 7 State Key Laboratory for Mechanical Behavior of Materials, Xi’an Jiaotong University (XJTU), Xi’an 710049, China

## Abstract

Coupling between lattice and order parameters, such as polarization in ferroelectrics and/or polarity in polar structures, has a strong impact on surface relaxation and reconstruction. However, up to now, surface structures that involve the termination of both matrix polarization and polar atomic planes have received little attention, particularly on the atomic scale. Here, we study surface structures on a BiFeO_3_ thin film using atomic-resolution scanning transmission electron microscopy and spectroscopy. Two types of surface structure are found, depending on the polarization of the underlying ferroelectric domain. On domains that have an upward polarization component, a layer with an Aurivillius-Bi_2_O_2_-like structural unit is observed. Dramatic changes in local properties are measured directly below the surface layer. On domains that have a downward polarization component, no reconstructions are visible. Calculations based on *ab initio* density functional theory reproduce the results and are used to interpret the formation of the surface structures.

The surfaces of perovskite oxides undergo surface relaxation^1^,^2^ and reconstruction^3^,^4^,^5^,^6^,^7^,^8^,^9^ owing to the breaking of translational symmetry. The resulting surface structures can have a strong influence on the functional properties of the materials when they possess large surface-to-volume ratio^4^,^10^. Surface structures also govern the performance of devices that rely on interfacial coupling or interactions^11^,^12^,^13^,^14^. Depending on the charges of the crystallographic termination planes, perovskite surfaces can be distinguished into two groups: polar surfaces with a net charge and non-polar surfaces without a net charge. The atomic, electronic and magnetic properties of such surfaces can differ significantly from one group to the other^1^,^6^,^7^,^8^,^9^,^15^,^16^,^17^,^18^. In ferroelectric oxides, surfaces can also be charged or uncharged depending on the orientation of the surface plane with respect to the spontaneous polarization ***P***_s_, resulting in a strong influence on surface structure and properties^19^,^20^,^21^. For instance, surface chemistry and surface adsorption/desorption behavior in important ferroelectrics such as BaTiO_3_ (BTO) and Pb(Zr,Ti)O_3_ (PZT) are highly dependent on ***P***_s_^19^,^21^. Although BTO and PZT are polarized along the tetragonal [001] axis at room temperature, it should be noted that the (001) surfaces of these structures, terminated by either a BaO or a TiO_2_ plane, are charge neutral.

There is another category of ferroelectrics that possesses ferroelectric polarization and polar atomic planes simultaneously but is little studied. A prototypical material is BiFeO_3_ (BFO), which exhibits both ferroelectric and G-type antiferromagnetic order at room temperature^22^. Although the multiferroic phase of BFO has a rhombohedrally-distorted *R3c* structure^22^, for convenience the crystallographic notation for a pseudocubic unit cell is used throughout the text unless specifically defined otherwise. As illustrated schematically in Fig. 1(a), the (BiO)^+^ and (FeO_2_)^−^ layers in BFO stack alternately along the <001> axis, leading to the formation of a polar {001} surface. In addition, off-center displacements of the Fe and O atoms with respect to the Bi sub-lattice result in a large ***P***_s_ of approximately 0.9–1.0 C/m^2^ 22 along the direction of the tensile-distorted [111] body diagonal. The polarization contains an <001> component that interacts with the charges on the terminating surface, as illustrated in Fig. 1. Depending on the polarization direction and the termination of the atomic planes, four different surface configurations can be obtained. Figure 1(b) shows all of these surface configurations, which are defined as type I to type IV, respectively. For type I and type IV surfaces, the charges that are caused by the [001] component of ***P***_s_ (i.e., ***P***_*z*_) and that of the surface polarity accumulate additively, while for type II and type III surfaces the charges are subtractive and can compensate. Interaction between the bulk polarization and the plane polarity is expected to have a significant influence on surface relaxation and reconstruction. The study of such surface phenomena is of great importance to gain a basic understanding of physical interactions between lattices, charges and polarizations at surfaces or interfaces in BFO, which may involve electrical screening and ferroelectric domain ordering^23^. These interactions will affect the local electronic, ferroelectric and magnetic properties of the material, with implications for potential applications such as exchange bias^24^ in interface-controlled devices for future nanoelectronics.

Here, we present an atomic-scale study of the surfaces of a BFO thin film grown on a DyScO_3_ (DSO) (110)_o_ substrate, where the subscript o refers to an orthorhombic lattice. By performing atomic-resolution scanning transmission electron microscopy (STEM) and spectroscopy, combined with first-principles density functional theory (DFT) calculations, we reveal two distinct surface structures, which depend on the polarization direction of domains in the film. By relying on an excellent match between the results of *ab initio* DFT calculations and our experiments, we determine the local ferroelectric and magnetic properties on the basis of atomic positions in the DFT-calculated model. The formation of the two types of surface structure is also discussed on the basis of polarization and the polarity of the surface plane.

## Results

### Structure and chemistry

**Figure 1:**
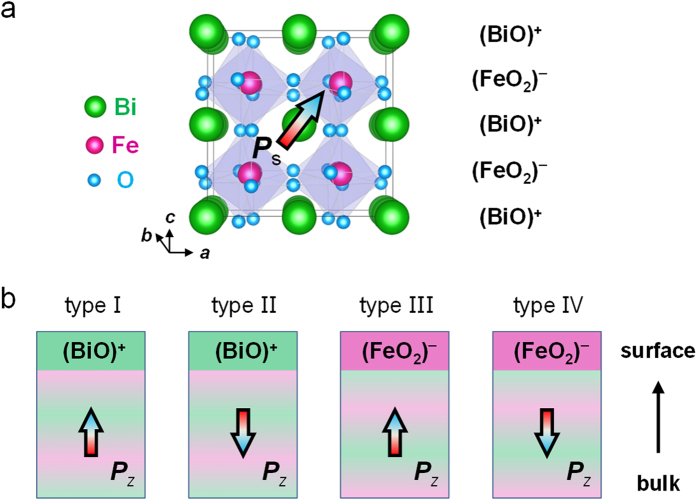
Schematic diagrams of the structure of a BFO multiferroic phase and four surface configurations. (**a**) 2 × 2 × 2 pseudocubic unit cells of room temperature BFO showing displacements of the Fe and O atoms along the [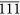] axis with respect to the Bi sub-lattice. The displacements lead to a spontaneous polarization ***P***_s_ pointing towards the [111] body diagonal. The (BiO)^+^ and (FeO_2_)^−^ atomic planes have positive and negative net charges and stack alternately along the [001] axis. Depending on the polarization direction and the termination of the atomic planes, four configurations of the (001) surface can be obtained. (**b**) Type I surface with an upward component of polarization and Type II surface with a downward component of polarization. Both surfaces are terminated by a polar (BiO)^+^ layer. Analogously, type III surface with an upward component of polarization and type IV surface with a downward component of polarization, both of which are terminated by a polar (FeO_2_)^−^ layer. The charges induced by the component of polarization (***P***_*z*_) and the charge of the polar surface termination planes are additive for type I and type IV surfaces, while they are subtractive and compensate for type II and type III surfaces.

**Figure 2:**
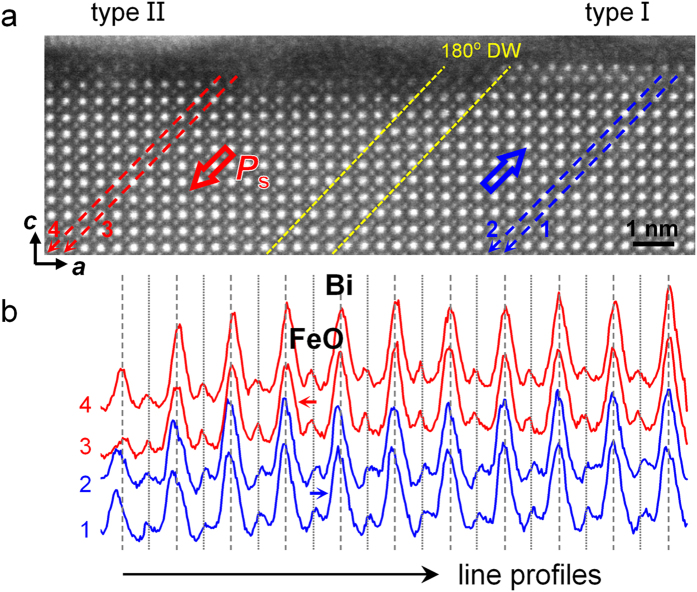
Two types of surface structure depending on the polarization of the film domains. (**a**) Atomic-resolution HAADF STEM image showing two BFO ferroelectric domains separated by a DW (marked by yellow dashed lines). The polarization vectors are marked in red for the left domain and in blue for the right domain. The surface structure on the left domain is distinguishable from that on the right domain. (**b**) Intensity line profiles generated from the left (red) and right (blue) ferroelectric domains, showing opposite shifts of the FeO columns with respect to the Bi columns. The dashed lines approximately trace the intensity peak positions of Bi, while the dotted lines are located in the middle of two adjacent dashed lines. The average distance between the Bi-Bi peaks is 0.395 nm.

Figure 2(a) shows a high-angle annular dark-field (HAADF) STEM^25^ image of a BFO (001) film on a DSO substrate, recorded along a <100> direction. In this image, the bright dots (i.e., peaks in intensity) correspond to heavy Bi atomic columns, while the less bright dots correspond to FeO columns. A signal from the pure O columns cannot be distinguished from the background. Along the film normal, the (BiO)^+^ and (FeO_2_)^−^ planes stack alternately, as in the perovskite structure (Fig. 1(a)). The chosen sample area contains two domains. A domain wall (DW), as marked by yellow dashed lines, can be traced by following a reversal in the shifts of FeO columns with respect to Bi columns. Figure 2(b) illustrates this reversal more clearly in the form of intensity profiles of the Bi and FeO columns, following the red lines in the left domain and the blue lines in the right domain in Fig. 2(a). The polarization vectors ***P***_s_ in the two domains are denoted by arrows in Fig. 2(a).

Corresponding to the two domains, the two types of surface structure are recognized in Fig. 2(a), which are separated by the DW. On the surface of the right domain, a double-atomic-layer (DL) is clearly visible, exhibiting image contrast that is similar to that of the BiO planes in the film matrix (see also an intensity plot in supplementary Figure S1). The two atomic layers have a lateral displacement of ***a*** <010>/2 with respect to one another. According to the direction of ***P***_s_, this surface corresponds to a type I surface, as defined in Fig. 1(b).

**Figure 3:**
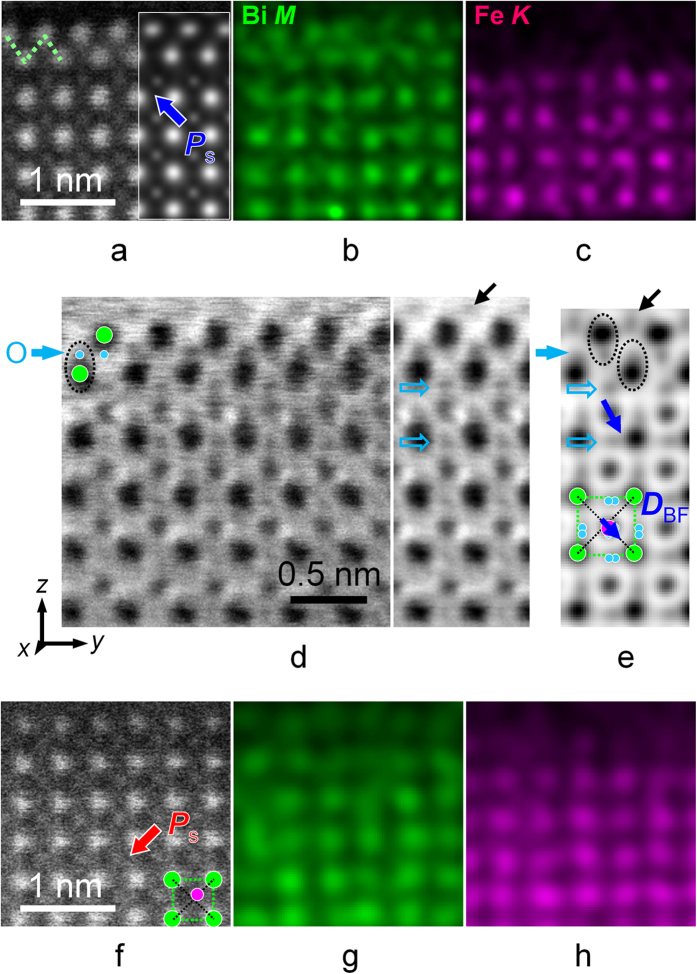
Determination of surface structure and chemistry. (**a**) Magnified HAADF STEM image showing atomic details in the double-atomic-layer on the type I surface. The inset shows a simulated image based on the DFT-calculated O8 model. (**b**) and (**c**) show atomic-resolution Bi *M* and Fe *K* EDXS maps, respectively, revealing a Bi double-layer on the surface of the BFO film domain, as indicated by the zigzag line in (**a**). (**d**) ABF STEM image showing the positions of O atoms in the vicinity of the Bi double-layer, providing evidence for a Bi-O structure unit of the Aurivillius phase. A laterally averaged image is shown on the right. (**e**) Simulated image calculated on the basis of the DFT-calculated O8 model, representing all of the surface features in (**d**). (**f**) Magnified HAADF STEM image and (**g**,**h**) corresponding EDXS elemental maps for Bi and Fe, showing atomic details on the type II surface, on which no Bi-O DL is visible.

The chemistry of the DL was revealed by using atomic-resolution energy-dispersive x-ray spectroscopy (EDXS) elemental mapping, as shown in Fig. 3(a–c). Figure 3(a) shows a magnified HAADF STEM image of the DL, where chemical mapping was performed. Figures 3(b,c) show Bi and Fe elemental maps, respectively, from which it is evident that the DL contains Bi atoms, while no Fe atoms are detected. The Bi DL has a zigzag configuration along the <100> direction (see dotted line in Fig. 3(a)), while it has a square pattern along the <110> direction (see supplementary Figure S2). The spacing between the layers was measured to be ~0.26 nm, which is consistent with that of Aurivillius-type layers formed in 0.95(Na_0.5_Bi_0.5_)TiO_3_−0.05BaTiO_3_ thin films^26^.

Structurally, an Aurivillius-type (Bi_2_O_2_)^2+^ layer contains an O atomic plane sandwiched between two Bi atomic planes (see supplementary Figure S3). Annular bright-field (ABF) STEM imaging^27^,^28^ was used to confirm its presence. As shown in Fig. 3(d), the ABF image on the left and its lateral average on the right reveal atomic columns as darker dots on a brighter background, including a signature from the expected pure O columns. A layer with relatively weak contrast (marked by a solid light blue arrow) can be attributed to the presence of an O atomic layer^29^. In this layer, an O atom (light blue) and a neighboring Bi atom (green) form a dumbbell-like configuration, as marked by dotted ellipses, which is reproduced by the simulated ABF image shown in Fig. 3(e). The simulation was calculated on the basis of an atomic O8 model obtained from first-principles DFT calculations (see Methods and supplementary Figures S4–S7). By using the same model, the HAADF image was also simulated and shown to have a good correspondence to the experimental image, as presented in the inset to Fig. 3(a). Based on these results, it is concluded that the DL structure is very close to that of the (Bi_2_O_2_)^2+^ unit in the Aurivillius phase.

The same analysis was applied to the surface structures on the left domain in Fig. 2(a). Figure 3(f) shows a magnified HAADF STEM image of the surface area. Although the intensities of the atomic columns decay slightly at the surface due to a reduction in specimen thickness, the atomic features remain well-resolved. Based on the atomic-resolution HAADF STEM image and EDXS maps shown in Fig. 3(g,h), the terminating plane on the surface is most likely to be a perovskite (BiO)^+^ plane. The (Bi_2_O_2_)^2+^-like structure that was observed on the surface of the domain with an upward ***P***_s_ component (i.e., a type I surface) is now absent. According to the polarization direction (i.e., a downward component of ***P***_s_), the surface of the domain can be classified as type II, as defined in Fig. 1(b).

### Lattice expansion

**Figure 4:**
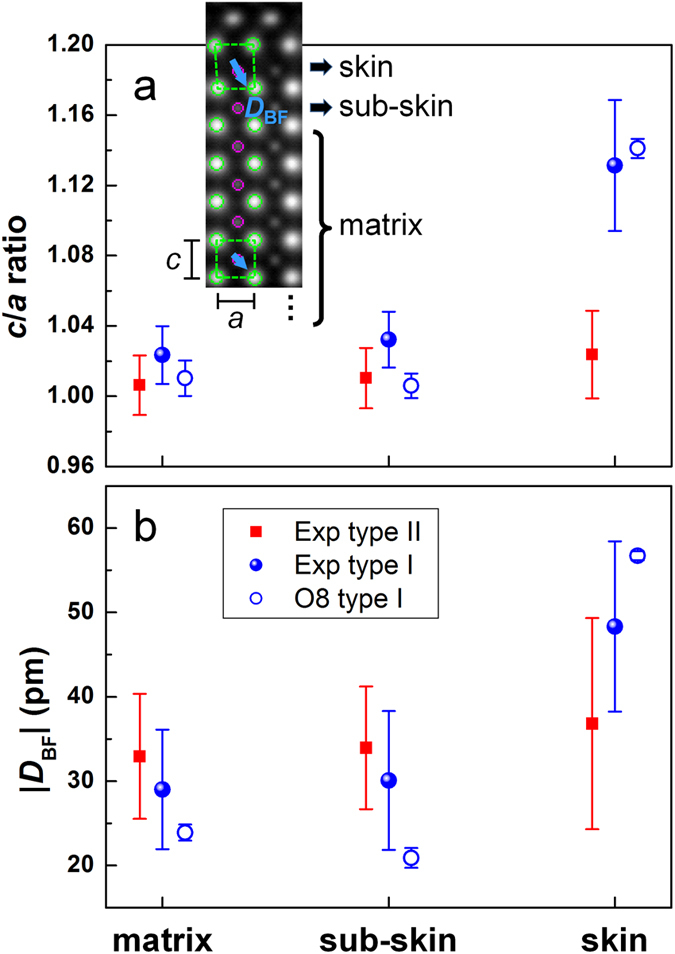
Measurements of unit cell tetragonality and off-center displacements of FeO columns. The experimental measurements (filled symbols) were calibrated using the lattice parameter of the DSO substrate (*a* = 0.3955 nm). (**a**) Tetragonality *c*/*a* for the film matrix, sub-skin and skin layers of BFO. Inset is a simulated HAADF image for the O8 model. (**b**) Moduli of displacements ***D***_BF_ of FeO columns with respect to the centers of mass of the surrounding four Bi atoms. The statistical error bars in the matrix are smaller because of the larger amount of data.

The lattice parameters in the surface regions were investigated by quantifying the atomic-resolution HAADF STEM images. The positions of intensity peaks corresponding to Bi atomic columns were determined from fast-acquisition HAADF STEM images by fitting two-dimensional Gaussian functions to the intensity peaks^30^. Based on the measured positions of the intensity peaks, the tetragonality (i.e., the *c*/*a* ratio) was calculated for each unit cell. Figure 4(a) shows the mean values of *c*/*a* and the corresponding standard deviations *σ* as error bars in the BFO film matrix, the sub-skin and skin layers (see the legend in the inset of Fig. 4(a)) for both the type I and the type II surface. In the BFO film matrix, for both types of domains the *c*/*a* ratio is nearly constant at a value of ~1.02. Considering the lattice mismatch of ~0.3% between DSO^31^ and BFO^22^, the measured tetragonality of 1.02 indicates that the BFO film is fully strained due to the epitaxial relationship with the DSO substrate. The *c*/*a* ratio remains almost unchanged from the matrix to the BFO skin layer for the type II surface area, while in the skin layer in the type I surface region this ratio increases abruptly to a value of ~1.13, which is much larger than the value for the BFO film matrix on the DSO substrate.

Accompanying the large *c*/*a* ratio in the BFO skin layer on the type I surface, the off-center displacements of the FeO columns with respect to the centers of mass of the surrounding four Bi atomic columns also show a dramatic increase, suggesting a strong coupling between the polarization and the lattice distortion. As shown in Fig. 3(d) and the inset to Fig. 4(a), in the BFO film matrix the central FeO columns (magenta) are shifted down and right along approximately the <01
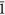
> diagonal direction, with an average displacement |***D***_BF_| of ~30 pm (Fig. 4(b)). This value is very close to a displacement of 33 pm for Fe atoms reported for BFO grown on a TbScO_3_ substrate with a lattice mismatch of <0.14%^32^. In contrast, the off-center displacements of the FeO columns in the BFO skin layer are ~50 pm, i.e., 67% larger than those in the film matrix. In addition, the displacement vector undergoes a reorientation towards the <00
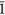
> direction. We also measure shifts of the O atoms, as marked by open arrows in Fig. 3(d,e), which do not exhibit a collinear relationship with the shifts of the FeO columns.

In the domain with the type II surface, no changes in off-center displacements of FeO columns were measured, consistent with the measured variation in *c*/*a* ratio in Fig. 4(a).

In order to understand the atomic structures and the large difference in lattice expansion in the skin layer between the two types of surface, we performed first-principles DFT calculations (see Methods), starting with free-surface relaxation of BFO (supplementary Figure S4). Although the calculation exhibits a different pathway for free-surface relaxation compared to our experimental observation, extremely large atom movements still occur in the vicinity of a type I surface. This behavior reveals a strong but natural demand to lower the system energy by ionic displacements, leading to a compensation of accumulated charges on this type of surface.

Based on the experimental results shown in Figs 2 and 3, supercells were constructed, comprising 2 × 2 × 6 cells of BFO together with a structural unit of Aurivillius or δ-Bi_2_O_3_ containing 8 O and 4 Bi atoms (i.e., a Bi_2_O_2_-like unit) at the surface as a reference model for a type I surface, in which the nominal valency of the full supercell is zero (see Methods and supplementary Figure S5). Considering the need to screen the positive charge induced by the matrix ***P***_s_ and the surface (BiO)^+^ layer at the type I surface (Fig. 1), the surface structure was tuned to electronegativity by deliberately introducing Bi vacancies or O adatoms to the reference model. The resulting models were named Bi*X* or O*X* (where *X* is an integer), corresponding to the residual Bi occupancy or the numbers of O adatoms at the surface (see Methods and supplementary Figure S6). Since it is very difficult to compare the total energies of two systems that contain different numbers of atoms, the *c*/*a* ratio of the BFO skin was used as a figure of merit for determining the best matching model for the type I surface. All of the relaxed structures are shown in supplementary Figure S6, alongside corresponding HAADF STEM image simulations. Our *ab initio* result confirms that, following an increase in negative surface charge in the Bi double-layer, the *c*/*a* ratio of the BFO skin increases accordingly in all of the relaxed models (supplementary Figure S7), in agreement with the experimental result and in accordance with the surface charge compensation mechanism. Furthermore, based on the good correspondence between the simulated and experimental HAADF STEM images (supplementary Figure S1), it is reasonable to conclude that the presence of outer surface O adatoms improves the stability of a type I surface. A closer look at the averaged ABF STEM image in Fig. 3(d) reveals weak contrast from O atoms at the outer surface, as indicated by the black arrow, which is reproduced well in the simulated image in Fig. 3(e) based on the relaxed O8 model only. Moreover, a comparison of the *c*/*a* ratio and the displacement vector ***D***_BF_ further confirms the match between the DFT calculations and the experimental measurements, as shown in Fig. 4. Based on these results, the relaxed O8 model is considered to be representative of the real structure.

### Properties of the skin layer

**Figure 5:**
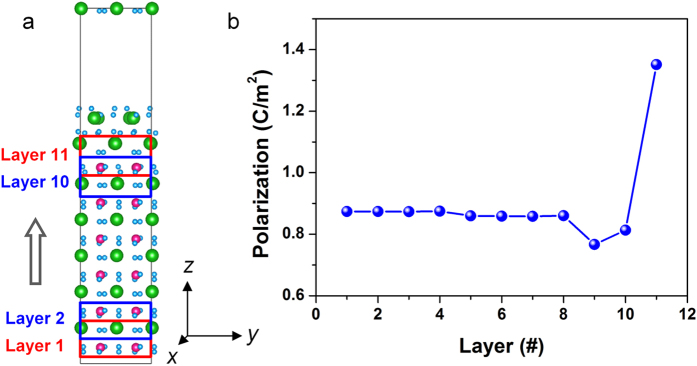
Variation in *P*_s_ from the matrix to the film surface based on *ab initio* calculations. (**a**) Schematic diagram for measuring the spontaneous polarization of the ‘sliding’ layers on the basis of O8 model. (**b**) Plot of polarization as a function of the ‘sliding’ layers. In the film matrix (i.e., from layer 1 to layer 8), the polarization remains constant at ~0.867 C/m^2^. It decreases slightly in the sub-skin unit cells, followed by an abrupt increase to ~1.4 C/m^2^ in the skin unit cell layer.

The spontaneous polarization of the surface region of the BFO film was calculated on the basis of the atomic positions in the O8 model shown in Fig. 5(a). The variation in polarization with distance from the film matrix to the surface was evaluated by using the ‘sliding’ unit cell method^33^. The polarization was calculated based on the equation: 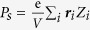, where e is the elementary charge and *V* is the unit cell volume^34^. The displacements ***r***_*i*_ for all of the ions from their centrosymmetric positions in the paraelectric phase were obtained from the O8 model. The Born charges *Z*_Bi_ = 4.37, *Z*_Fe_ = 3.49 and *Z*_O_ = −2.61 for the *R3c* structure were taken from Ref. 34. Figure 5(b) shows the modulus of ***P***_s_ plotted as a function of the ‘sliding’ BFO unit cell layer. In the film matrix (i.e., from layer 1 to layer 8), |***P***_s_| remains constant at ~0.867 C/m^2^. This value is close to a prediction of 0.873 C/m^2^ 34. The polarization decreases slightly in the sub-skin unit cells, followed by an abrupt increase to ~1.4 C/m^2^ in the skin unit cell layer, which is very close to that reported for the tetragonal phase^35^. The slight drop in the sub-skin layers arises mainly from a decrease of the in-plane components of ***P***_s_. From the model, we note that the in-plane components of polarization in the skin unit cell are comparable with that in the matrix. The increase in the out-of-plane component results in a rotation of the polarization vector in the skin unit cell layer in the <110> plane. This phenomenon is similar to the M_A_ phase transformation of BFO^36^, suggesting a similar relationship between the bound charge and the lattice strain.

According to the DFT calculations, the reconstruction in the surface layer also affects the local magnetic properties of BFO. The results of our calculations show that BFO still stabilizes in a G-type antiferromagnetic structure in both the type I and the type II surface. This is expected, considering the fact that the magnetic G-type to C-type order transition for BFO films occurs at a *c*/*a* ratio of ~1.19 as a result of an increase in compressive lattice strain^37^. The calculated Fe magnetic moment in the ground state decreases from ~3.8 μ_B_ in the strained film matrix to ~3.0 μ_B_ in the skin unit cell layer for the type I surface. In contrast, the magnetic moment remains at 3.8 μ_B_ for all of the Fe ions in the unreconstructed type II surface, which is comparable to the value of 4.11 ± 0.15 μ_B_ in a BFO single crystal^38^.

## Discussion and Conclusions

Our results reveal the detailed atomic structure in the surface region of a BFO film on a DSO substrate. Two types of surface structure exist, depending on the polarization direction of the underlying ferroelectric domains and the termination of the polar atomic planes at the surface. A structure similar to the Bi_2_O_2_ unit of the Aurivillius phase is found on the surface of film domains that have an upward component of ferroelectric polarization (type I surface). The same structure may be present in an image shown in Ref. 23 for a BFO film grown on a different substrate with a different thickness by molecular beam epitaxy, although it was not mentioned in that paper. Our observations are also numerically consistent with a BFO surface structure viewed along the <110> direction grown on a 5 nm La_0.7_Sr_0.3_MnO_3_/SrTiO_3_ substrate^39^, although the surface contraction layer was then attributed to the formation of a BFO dead layer instead of Bi_2_O_2±*δ*_. These findings indicate a general tendency of BFO film surfaces to reconstruct into this kind of structure.

The fact that the appearance of the two surface structures strongly depends on the polarization of the film domains implies a coupling between the structure and surface charges triggered by ferroelectric polarization. For the type I surface, the structural unit of the Aurivillius phase acts as a reservoir for negative charges from the excess of O in the outer Bi plane, which stabilizes the surface structure. The negative charges contribute to the compensation of positive charges associated with the upward component of polarization and the (BiO)^+^ polar surface termination. In contrast, the negative surface bound charges induced by the downward component of ***P***_s_ on the type II surface can be self-compensated by the positive charges of the (BiO)^+^ termination layer and therefore do not require an additional Bi_2_O_2±*δ*_ structure. Indeed, free surface relaxation of the R6 structure shows that only small changes in O column splitting from 52 to 76 pm in the {100} projected plane (marked by orange arrows in supplementary Figure S4) are needed to stabilize the structure of a type II surface. This change is presently below the detection limit of the applied experimental techniques.

Recently, Gao *et al*.^21^ studied the surface of a ferroelectric PZT film. In their work, a charged 180° DW and suppressed polarization were found at negatively polarized surfaces (i.e., with ***P***_s_ pointing opposite to the film surface), while no reconstruction was detected at positively polarized surfaces (i.e., with ***P***_s_ pointing towards the film surface). These observations were interpreted in terms of surface screening, possibly due to the presence of O vacancies on the former surface and Pb vacancies on the latter surface, controlled by ferroelectric ***P***_s_ alone. In the case of a BFO film, due to differences in crystal structure, chemistry and stacking polar atomic planes, we observe a different reconstruction that depends in an opposite way on polarization. A prominent reconstruction then occurs at a positively polarized type I surface, while no reconstruction is detected for a negatively polarized type II surface. Our results provide clear evidence for the combined contributions of ferroelectric polarization and polar atomic plane termination. These contributions can be considered in terms of a capability to alter the surface atomic structure in a different way from that for a PZT film, which is controlled by ***P***_s_ only. Based on our results and discussions, it is reasonable to expect an analogous behavior for surface reconstructions in other ferroelectric oxides with odd valences of cations, such as LiNbO_3_.

A large *c*/*a* ratio (~1.25) and an enhanced value of |***P***_s_| (~1.5 C/m^2^) in a tetragonal-like phase of BFO have already been reported^35^. This phase was found to be stabilized together with a rhombohedral-like phase by large compressive strain. For the rhombohedral-like phase, different *c*/*a* ratios were reported from 1.05 to 1.07^35^,^36^. In addition, Beekman *et al*. reported an S′ phase with a *c*/*a* value of 1.09^40^. Our study shows that the BFO skin unit cell layer has a comparable *c*/*a* ratio and magnetic ordering to the S′ phase, but possesses a very large value of ***P***_s_, as in the tetragonal-like phase. Our results provide an important structural basis for tuning the local multiferroic properties of BFO by controlling electrical boundary conditions.

## Methods

### Sample preparation

An epitaxial BiFeO_3_ thin film was grown on an epi-polished single crystalline DyScO_3_ (110)_o_ substrate (CrysTec GmbH, Berlin) by using pure-oxygen pressure radio frequency magnetron sputtering. This low-energy technique allows thin films with very high atomic structural perfection to be produced^41^. Prior to deposition, the chamber was pumped down to a background pressure below 1 × 10^‒5^ mbar. The substrate was then heated to the deposition temperature of 650 °C in an oxygen atmosphere at 2 mbar, which was maintained throughout film growth. Owing to the reduced loss of volatile Bi in the high oxygen condition, a stoichiometric BiFeO_3_ target was used. The target was pre-sputtered for several minutes to remove possible contamination on the surface. Layer deposition lasted for 3 hours. After growth, the sample was cooled naturally to room temperature.

Cross-sectional lamellar specimens for STEM investigations were prepared by focused ion beam (FIB) milling using an FEI Helios Nanolab 400 s dual-beam system^42^. The lamellae were cut from two directions, <100> and <110>, to investigate the atomic details systematically. The as-prepared lamellae were carefully thinned using 2.5 kV Ar ion milling in a Bal-Tec Res-120 system, followed by final cleaning with a 500 eV Ar ion beam (Fischione Nanomill, Model 1040) to remove possible damaged layers introduced during the previous milling procedures.

### Scanning transmission electron microscopy and image simulations

HAADF STEM imaging^25^ was carried out in an FEI Titan G3 60–300 “PICO” microscope equipped with a high-brightness field emission gun (XFEG), a monochromator unit, a probe spherical aberration (C_s_) corrector and a combined image C_s_-C_c_ (chromatic aberration) corrector^43^. ABF STEM imaging^27^ was performed on a JEOL JEM-ARM200F probe-C_s_-corrected microscope with a built-in ABF attachment. Atomic-resolution EDXS mapping was performed on an FEI Titan G2 80–200 ChemiSTEM microscope equipped with an XFEG, a probe C_s_ corrector and a super-X EDXS system^44^. All of the microscopes were operated at 200 kV. The convergence semi-angle for STEM imaging was approximately 22 mrad, while the collection semi-angle was 12–24 mrad for ABF imaging and 70–176 or 200 mrad for HAADF imaging. All high-resolution ABF/HAADF images shown in this work are shown as raw data without any post-filtering.

Multislice ABF and HAADF STEM image simulations were performed using Dr. Probe software^45^ on the basis of atomic models relaxed by first-principles DFT calculations and using experimental imaging parameters. Considering the unique site occupation in the Bi75, Bi25 and O4 models (caused by the size of the supercell), surface Bi disordering was added manually to obtain a correct surface intensity. However, this operation did not change the simulated details in the BFO skin layer. A series of sample thicknesses (up to 60 nm in steps of approximately 0.8 nm, i.e., two BFO pseudocubic unit cells) was tested. A thickness of 38–40 nm was finally utilized by matching the image contrast in the experimental and simulated images. The sample misalignment angle and the aberrations of the microscope were set to zero for the simulations. Structural models were visualized using VESTA software^46^.

### Ab initio simulations

First-principles DFT calculations within the local spin-density approximation (LSDA)^47^,^48^ were performed using the Vienna *ab initio* Simulation Package (VASP)^49^,^50^. All results were obtained using the projector-augmented plane-wave method^51^,^52^ by explicitly treating the valence electrons as follows: 15 for Bi (5*d*^10^6*s*^2^6*p*^3^), 14 for Fe (3*p*^6^3*d*^6^4*s*^2^) and 6 for O (2*s*^2^2*p*^4^). The calculations do not include spin-orbit corrections. Brillouin zone integrations were performed with Gaussian broadening^53^ of 0.05 eV during all relaxations. Bulk calculations were performed with a 5 × 5 × 5 Monkhorst-Pack ***k***-point mesh^54^ centered at Γ and a 550 eV plane-wave cutoff, both of which result in good convergence of the computed ground-state properties. Structural optimizations were achieved by allowing the atoms in the unit cell to relax until all of the forces on each atomic site were below 5 meV/Å and simultaneously achieving a total energy convergence of 10^−6^ eV. In order to correct for the metallic behavior observed in the LDA band structure, we applied the LDA + U approach described by Dudarev *et al*.^55^, in which only an effective Hubbard parameter U_eff_ = U − J enters the Hamiltonian. The magnitude of U_eff_ was varied between 0 and 7 eV for the Fe *d* states (the standard LSDA result corresponds to U_eff_ = 0 eV). The effective Hubbard parameter U_eff_ = 2 eV was used in all of our calculations. Higher values of the U_eff_ parameter also yielded similar results for the structural properties.

The ground state of bulk BFO is *R3c* and exhibits G-type antiferromagnetism^22^. However, strain from the substrate is a key factor that can be used to tune its phase, as pointed out in recent studies^56^. In our case, the use of a DSO substrate lowers the symmetry of the BFO. We therefore used an orthogonalized coordinate system with *P1* symmetry. We first transformed the experimental BFO coordinates (ICSD No. 109370, from Ref. 57) to the orthogonalized coordinate system by using CellMuncher.exe included in the Dr. Probe software package^45^. A 2 × 2 × 2 supercell containing 40 atoms of BFO was chosen, in order to accommodate all possible antiferrodistortive rotations of the FeO_6_ octahedra and to enforce the experimental triclinic and monoclinic geometries for the different phases. We obtained a relaxed BFO bulk cell of 40 atoms with *a* = 0.7791 nm, *b* = 0.7785, *c* = 0.7784 nm and *α* = *β* = *γ* = 90°. A schematic diagram is shown in Fig. 1 in the main text. It should also be noted that the optimized lattice constants are underestimated with respect to experimental values as a consequence of using the LSDA, with a scaling factor of ~1.02 that is consistent with Ref. 58.

In order to simulate lattice strain imposed by the DSO substrate, we compressed the lattice constant along the ***x***- and ***y***- axes and fixed them to the calculated lattice constant of DSO (110)_o_. We then scanned along the ***z***-axis and fully relaxed all of the atoms to obtain a local energy minimum. This approach created a pseudocubic phase with *a* = *b* = 0.773 nm and *c* = 0.7877 nm. The resulting *c*/*a* ratio is 1.02, which is in good agreement with our experimental observations. It also gives a ground state Fe magnetic moment of 3.8 μ_B_, which is slightly lower than the value of 4.0 μ_B_ in the *R3c* phase. Such a pseudocubic unit cell was used to build the surface structure.

In order to construct a Bi-terminated surface, a 6-layer 2 × 2 supercell with BiO-terminated surfaces (denoted R6) was constructed as a reference system (supplementary Figure S4). An additional 1.5 nm layer of vacuum was used to exclude possible interactions between the two surfaces and a dipole interaction correction was applied perpendicular to the surface in all surface calculations. One layer of eight O atoms and one layer of four Bi atoms in an Aurivillius type configuration (supplementary Figures S5(c1) and Bi100/O0 model in supplementary Figure S6) were added on top of the R6 structure to simulate the atomic behavior observed experimentally. This model acts as a starting point, because the nominal valency of the system is 0. Switching the 8 O atoms on the sites between the Bi-Bi interlayer (Aurivillius-like configuration in supplementary Figure S5(c1)) and the outermost layer (δ-Bi_2_O_3_-like occupancy in supplementary Figure S5(c2,c3)) showed little influence on the *c*/*a* ratio, with a system energy for the latter case at least 0.75 eV higher than for the former case. Therefore, site selections were not considered in the following calculations.

Charge compensation was considered by removing Bi or adding O atoms on the basis of the Bi100/O0 model. As shown in detail in supplementary Figure S6, Bi75, Bi50 and Bi25 models were constructed by removing 1, 2 or 3 surface Bi atoms, while O1 to O8 models were constructed by adding 1 to 8 O atoms on the outer Bi surface on the basis of the Bi100/O0 model. Relaxation of the structures was carried out by fixing the atoms in the bottom 3 layers of the BFO and fully relaxing the other atoms. Convergence was obtained using a 3 × 3 × 1 Monkhorst-Pack special ***k***-point grid with a cutoff energy of 550 eV, in order to terminate the plane-wave expansion. The criterion for relaxation convergence was the same as for the bulk calculations.

## Additional Information

**How to cite this article**: Jin, L. *et al*. Surface reconstructions and related local properties of a BiFeO_3_ thin film. *Sci. Rep.* **7**, 39698; doi: 10.1038/srep39698 (2017).

**Publisher's note:** Springer Nature remains neutral with regard to jurisdictional claims in published maps and institutional affiliations.

## Supplementary Material

Supplementary Information
