# Supplementary material for: Surface reconstructions and related local properties of a BiFeO3 thin film
Source: Sci Rep. 2017 Jan 19;7:39698. doi: 10.1038/srep39698 (PMC5244358; doi:10.1038/srep39698)
Supplement: Supplementary Information [file srep39698-s1.pdf]

Supplementary information for

Surface reconstructions and related local properties of a $BiFeO_3$ thin film

L. Jin,[1,2,*] P. X. Xu,[3] Y. Zeng,[1,4,5] L. Lu,[4] J. Barthel,[2,6] T. Schulthess,[3] R. E. Dunin-Borkowski,[1,2] H. Wang,[4,7] C. L. Jia,[1,2,4,7]

[1]Peter Grünberg Institute, Research Centre Jülich, D-52425 Jülich, Germany

[2]Ernst Ruska-Centre for Microscopy and Spectroscopy with Electrons, Research Centre Jülich, D-52425 Jülich, Germany

[3]Institute for Theoretical Physics, ETH Zurich, 8093 Zurich, Switzerland

[4]The School of Electronic and Information Engineering, Xi'an Jiaotong University (XJTU), Xi'an 710049, China

[5]State Key Lab of New Ceramics and Fine Processing and School of Materials Science and Engineering, Tsinghua University, Beijing 100084, China

[6]Central Facility for Electron Microscopy, RWTH Aachen University, D-52074 Aachen, Germany

[7]State Key Laboratory for Mechanical Behavior of Materials, Xi'an Jiaotong University (XJTU), Xi'an 710049, China

[*]*E*-mail: l.jin@fz-juelich.de

Supporting figures

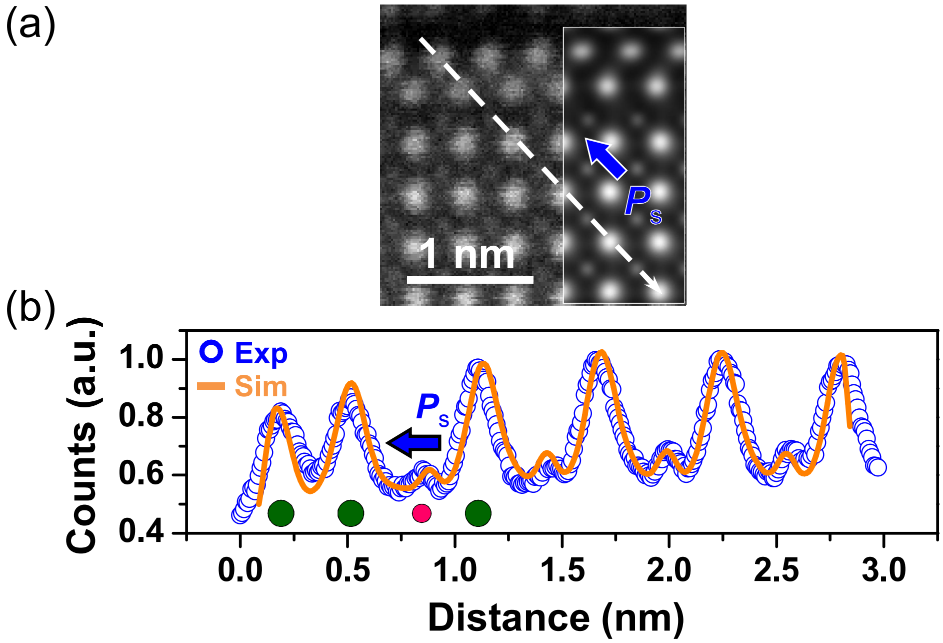

Figure S1 Comparison between experimental and simulated image based on a DFT-relaxed O8 model. (a) Atomic-resolution HAADF STEM image and simulated image. (b) Normalized intensity profile along the directions indicated by the dashed lines in (a), showing consistent modulations of a Bi (green) and an FeO (magenta) signal between the experimental and simulated images.

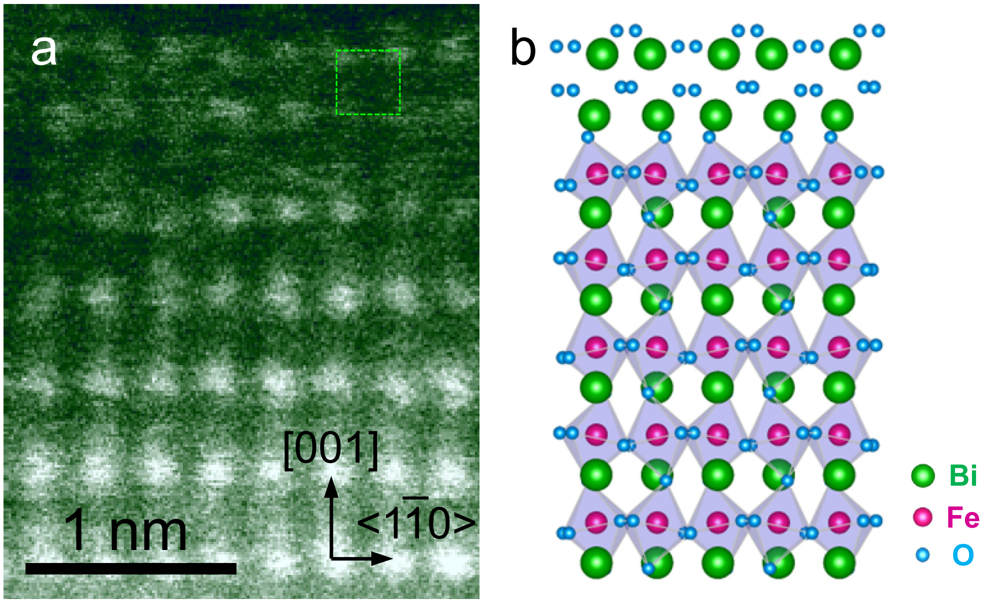

Figure S2 Atomic details of a Bi-O double-layer projected along the <110> pseudocubic axis. (a) HAADF STEM image showing the projection of an Aurivillius-like structure along the <110> direction. The Bi double-layer forms a square pattern, as indicated by the green rectangle. (b) Atomic O8 model calculated using a first-principles DFT calculation, viewed along the same orientation.

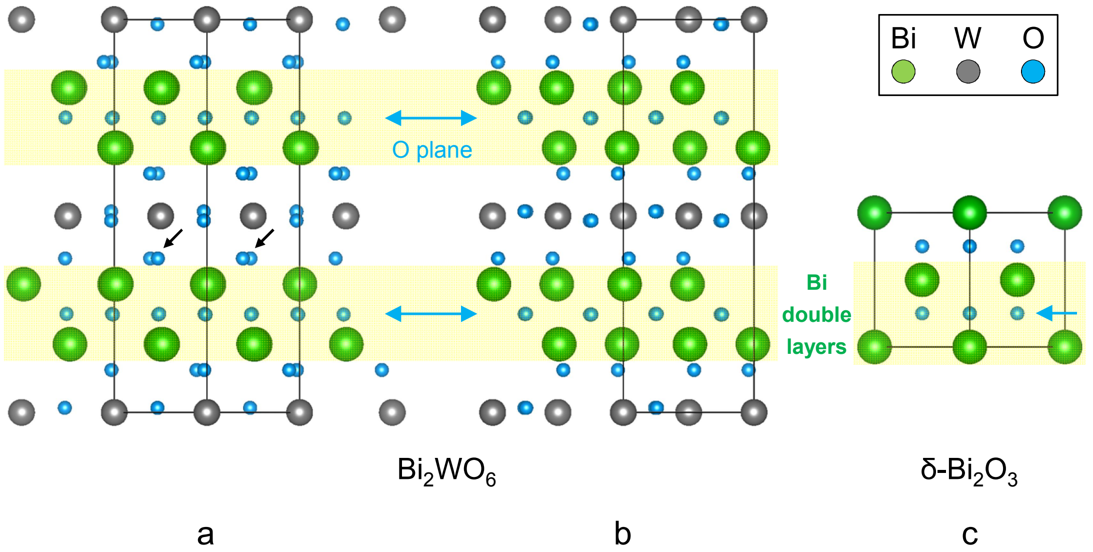

Figure S3 Crystal structure of a Bi_2WO_6 Aurivillius phase [1], including a structural unit of double Bi-O layers. (a) [110] projection and (b) [100] projection of the Bi_2WO_6 structure. An O atomic plane (marked by arrows) is located between two Bi atomic planes, as highlighted in yellow. Such a configuration is similar to the structure of δ-Bi_2O_3 [2], in which 25% of the O sites are vacant. (c) Structure of δ-Bi_2O_3 projected along the [110] direction.

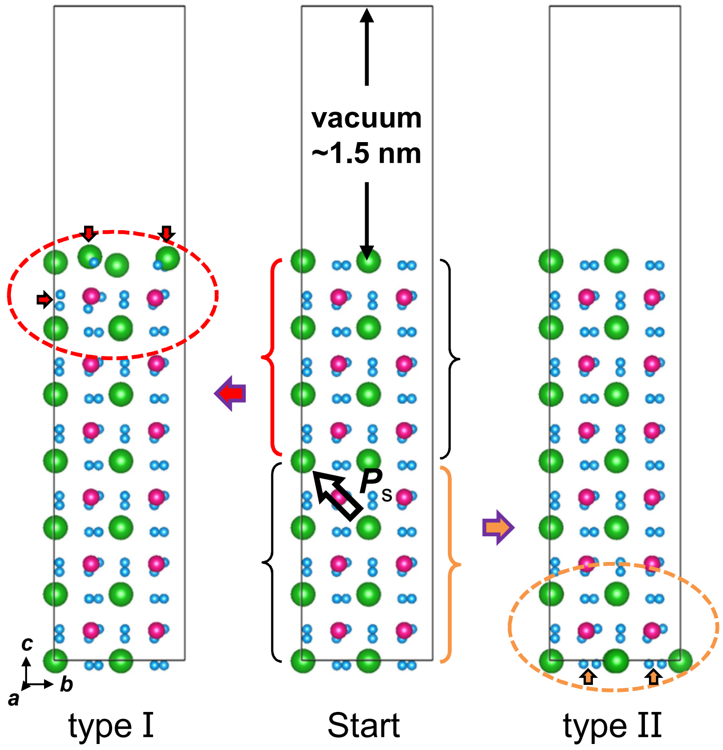

Figure S4 Free surface relaxation of strained $BiFeO_3$ (*c*/*a* =1.02) predicted by *ab initio* calculations. Central panel: starting supercell R6 structure (*a* = *b* = 0.773 nm, *c* = 3.865 nm), consisting of 2×2×6 BFO pseudocubic unit cells and ~1.5 nm of vacuum along the ***c***-axis in order to exclude possible interactions between the top and bottom surfaces. The Fe/O atoms are displaced along the $[11\bar{1}]$ axis with respect to the Bi lattice; correspondingly, $\boldsymbol{P}_s$ points towards the $[\bar{1}\bar{1}1]$ direction. Relaxation of the structure was carried out by fixing the atoms in the 3 layers enclosed by black brackets and fully relaxing the other atoms. Left panel: Type I surface relaxation marked by a red bracket. Right panel: Type II surface marked by an orange bracket. Prominent atomic shifts are evident in the vicinity of the type I surface (enclosed by a red circle). In contrast, only a tiny O splitting from 52 to 76 pm occurs in the ***bc*** plane on the type II surface (enclosed by an orange circle), providing evidence for a strong demand to lower the energy at a charge-accumulated type I surface, when compared to a self-compensated type II surface.

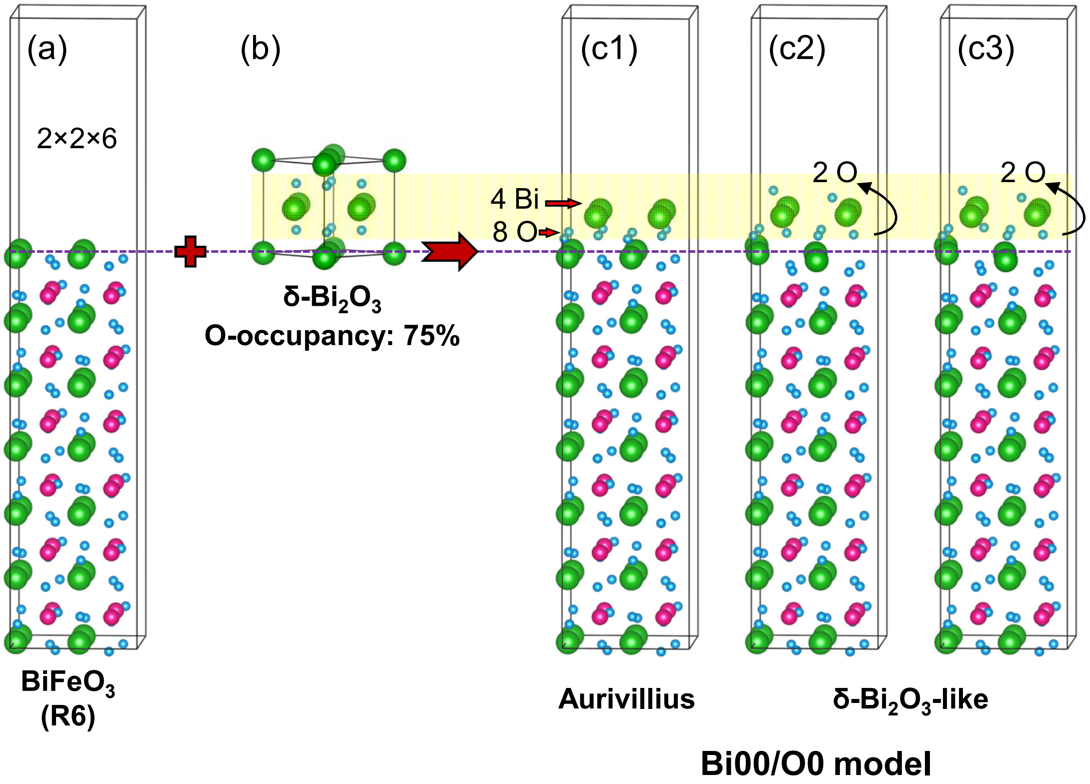

Figure S5 Construction of a Bi100/O0 supercell. (a) and (b) show the BFO R6 and δ-Bi_2O_3 components used to construct a supercell. (c1)-(c3) show three different models by considering the atomic site occupation of 8 O for (c1) an Aurivillius-like geometry, i.e., with 8 O located in the Bi-Bi interlayer (fully occupied) and (c2) and (c3) δ-Bi_2O_3-like occupation, i.e., with only 6 O located on the 8 atomic sites of the Bi-Bi interlayer. After relaxation, the *c*/*a* ratio is essentially the same, with the δ-Bi_2O_3-like occupation possessing much higher system energy (by at least 0.75 eV) than the Aurivillius-like configuration.

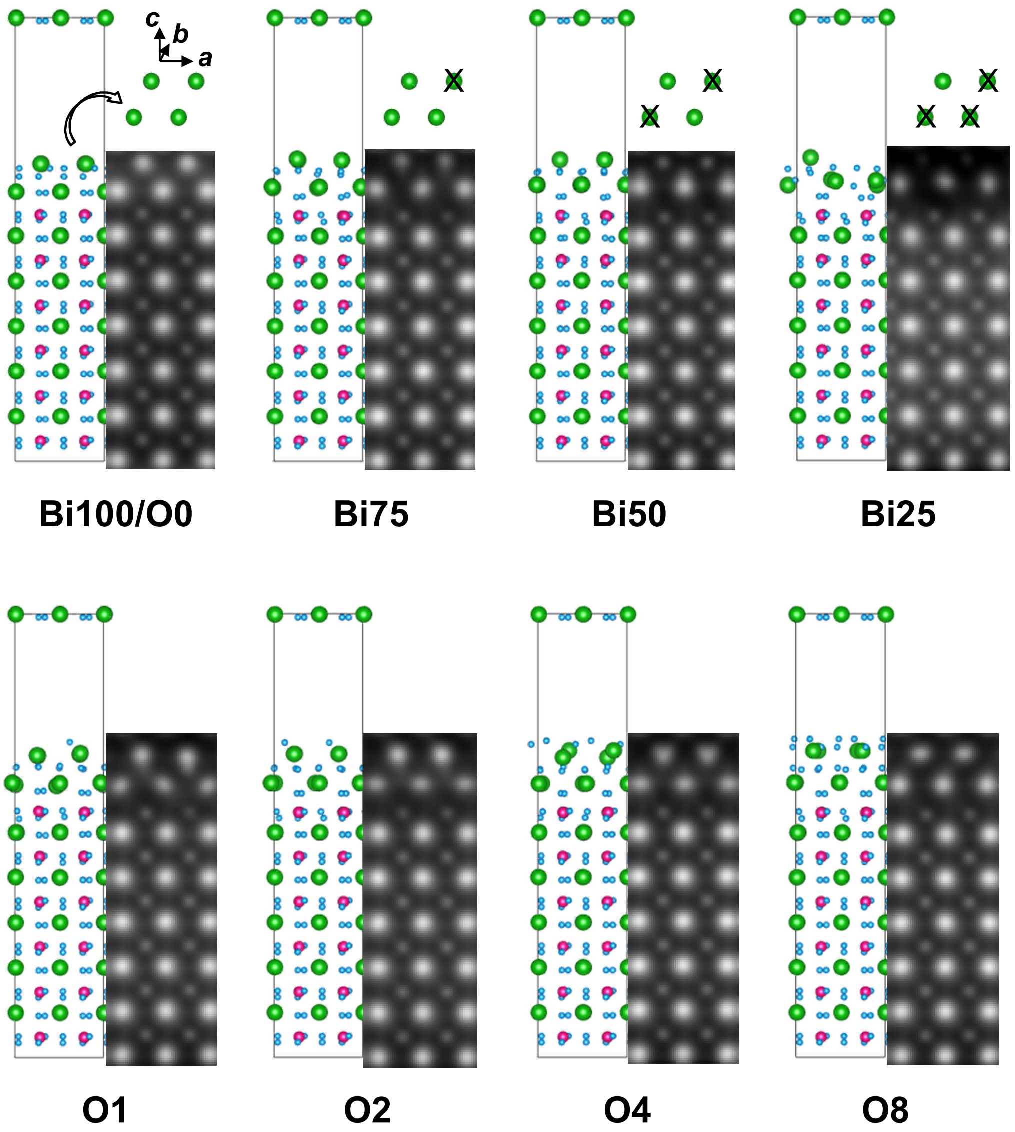

Figure S6 Relaxed atomic models and corresponding HAADF STEM simulated images. The construction of a Bi100/O0 model was described in supplementary Figure S5 for an Aurivillius-like surface geometry. Bi75, Bi50 and Bi25 models were constructed by removing 1, 2 and 3 surface Bi atoms (marked by crosses) on the basis of the Bi100/O0 model. O1 to O8 models were constructed by adding 1 to 8 O atoms on the outer Bi surface of the Bi100/O0 model.

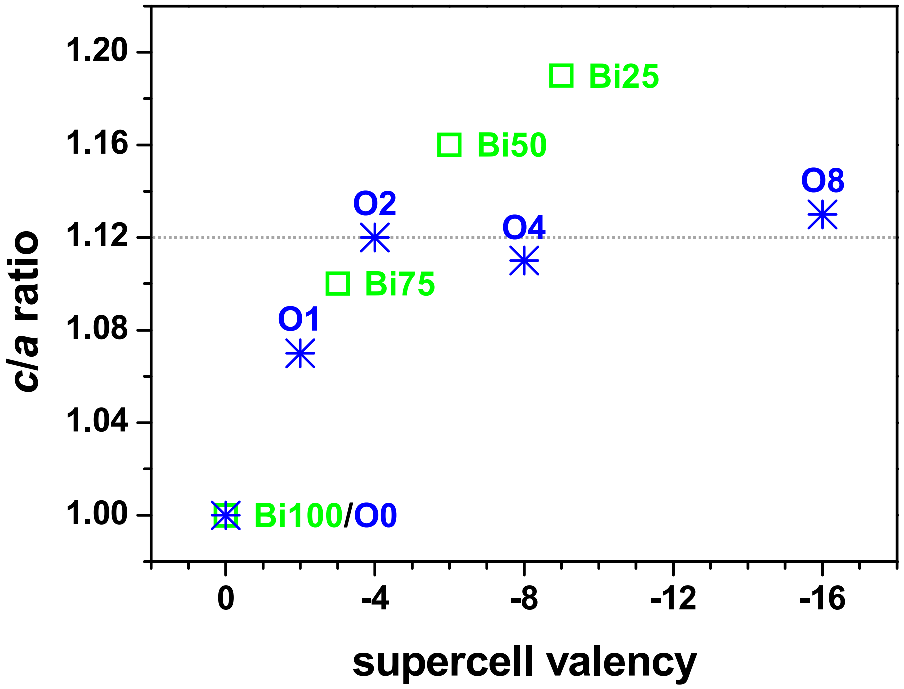

Figure S7 Plot of the calculated *c*/*a* ratio as a function of the net valency of the entire supercell. The *c*/*a* ratio was calculated on the basis of simulated HAADF images, as shown in supplementary Figure S6. In order to ensure measurement accuracy, the sampling rate was set to 0.0086 nm for the simulation, which is identical to the experimental value shown in Figure 2(b) in the main text. A plateau is present for the O2-O8 models, indicating saturation of negative charge compensation for O.

Supplementary references:

[1] Deniz, H. *et al.* Nanoscale Bi_2FeO_{6-x} precipitates in $BiFeO_3$ thin films: a metastable Aurivillius phase. *J. Mater. Sci.* **49**, 6952-6960 (2014).

[2] Zhong, G., Wang, Y., Dai, Z., Wang, J. & Zeng, Z. Oxygen vacancy configuration of δ-Bi_2O_3: an *ab initio* study. *Phys. Status Solidi B* **246**, 97-101 (2009).
